# Single-Machine Scheduling with Multi-Criteria and Due- Windows

**DOI:** 10.12688/f1000research.173324.1

**Published:** 2026-01-12

**Authors:** Dhuha Athal Mohsin, Hussam Abid Ali Mohammed

**Affiliations:** 1 Department of Mathematics, University of Kerbala, College of Education for Pure Sciences, Karbala, Iraq

**Keywords:** Due-Windows, Earliness, Tardiness, Late of Work, Multi-Criteria, Single-Machine Scheduling, Efficient Solution, Dominance Rules, Just-in-Time Systems

## Abstract

This paper investigates the Single-Machine Scheduling Problem under a multi-criteria optimization framework with due-window constraints, a class of NP-hard problems. The research introduces a mathematical formulation that integrates three performance measures—maximum earliness, maximum tardiness, and maximum late work—into a unified scheduling model. The due date of each job is represented as a flexible time interval bounded by minimum and maximum limits, providing a more realistic representation of production and service systems.

To address the complexity of the problem, several special cases are analyzed, and a set of dominance rules is proposed to identify efficient sequences that minimize computational effort while maintaining optimality. These rules extend classical sequencing principles such as the Minimum Slack Time and Earliest Due Date rules to multi-objective contexts. So, in Results and Discussion the comparative analysis demonstrates that the proposed dominance-based approach provides reliable and efficient solutions consistent with those obtained by the Complete Enumeration Method (CEM), but with significantly reduced computational complexity.

The theoretical contributions of this study offer a solid foundation for understanding trade-offs between different scheduling criteria within due-window environments. Practical implications arise in Just-in-Time (JIT) systems, where minimizing both early and late job completions directly impacts cost efficiency and workflow stability.

Although the analysis is restricted to a deterministic single-machine setup, the proposed framework can be extended to multi-machine, stochastic, or dynamic scheduling environments. Future research may focus on developing heuristic and metaheuristic algorithms to solve larger instances efficiently. Overall, the study contributes to the advancement of multi-criteria scheduling theory by combining mathematical rigor with practical relevance for real-world manufacturing and service operations.

## Introduction

In scheduling theory, the concept of a due-window represents a flexible time interval during which jobs can be completed without incurring penalties. Any job finished before the start of this window is considered early and penalized, while jobs completed after its end are regarded as tardy and also penalized. This framework provides a more realistic model than fixed deadlines, as it reflects practical manufacturing and service environments where customers or suppliers often allow a tolerance range for delivery. The considered problem belongs to the types of scheduling problems that defined according to due dates. ^1^ The Just-in-Time (JIT) approach in production planning and control aims to minimize waste and reduce inventory levels to zero. ^2^ Within this philosophy, finishing jobs either earlier or later than the assigned due date leads to extra costs, which are viewed as inefficient. Hence, in a JIT environment, it is preferable to complete jobs inside their specified due date windows. ^3^

In Ref. 4, scheduling problems under due-window settings was examined. Some studies assume that both the location and size of the due-window are predetermined, while others treat them as decision variables that must be optimized. Also others discussed the common due windows for single machine scheduling problem in Ref. 5. Problems of the latter type are referred to as due-window assignment problems. Extensive reviews of outcomes related to due date assignment problems are presented in Ref. 6, as well as Refs. 4 and 7. For instance, survey ^4^ discusses general formulations where earliness and tardiness are modeled as arbitrary non-decreasing functions, as examined by Refs. 8– 10. The concept of scheduling problems with due-windows is introduced by Ref. 11. More recent research on scheduling under common or flexible due-windows can be found in studies conducted by Refs. 12– 15.

In these cases, penalties may result not only from earliness and tardiness but also from delaying the start of the window or enlarging its size. For example Ref. 16 proposed a polynomial-time solution for the single-machine due-window assignment problem, where both the position and the size of the window are optimized simultaneously. Earlier Ref. 17 investigated models in which either the starting point or the finishing point of the window is adjustable, with the objective of minimizing the total penalties associated with earliness, tardiness, and the window size. In a related study, ^18^ focused on cases where the window size is fixed and sought to determine its best position to reduce the weighted sum of penalties.

In Ref. 19, this study investigates single-machine due-window assignment under common and slack windows, incorporating variable processing times, delivery times, and the possibility of outsourcing (job rejection). The authors prove polynomial solvability (specifically an $O\left(n^{6}\right)\;$ algorithm) for these enhanced models, and validate their approach with computational experiments showing improvements in resource utilization, cost reduction, and production efficiency.

In Refs. 18 and 20, the authors investigated the assignment of a common due-window for all jobs in single-processor and parallel-processor scheduling problems, respectively. Their approach assumes that the size of the due-window is predetermined. The studies in Refs. 16, 21 and 22 extended this model by treating both the position and the size of the due-window as decision variables. Furthermore, Ref. 23, as well as Ref. 24, examined related problems that incorporate an additional flow-time penalty. Only Ref. 25, together Ref. 26, have addressed scheduling on single and parallel processors with due-window assignment under a general min–max type criterion.

In Ref. 27, this paper addresses the problem of assigning a common due date or due window to all jobs, with objective to minimize total weighted early and late work. The authors derive that when the window or due date is unbounded, or when window length is unbounded, the problem admits efficient algorithms O(nlogn) or even linear time), but becomes NP-hard when these bounds are imposed.

The problem of multi-criteria optimization has been extensively studied in the literature. In Ref. 28 proposed a Multi-Objective Bat Algorithm (MOBA) for solving nonlinear programming problems involving multiple conflicting objectives. Their findings revealed superior performance and convergence of MOBA compared to other evolutionary optimization methods. In another study, ^29^ developed approaches to obtain exact and near Pareto-optimal solutions in scheduling problems with the aim of minimizing total completion time and total late work. These approaches achieved a balance between solution accuracy and computational efficiency. Ref. 30 introduced dominance rules for single-machine scheduling problems with weighted multi-criteria objectives, which improved solution quality while reducing computational effort by eliminating non-optimal schedules. For Ref. 31 investigated the dual objectives of maximizing early job times and minimizing the range of lateness, proposing both exact and heuristic algorithms that demonstrated efficiency and accuracy in handling complex single-machine scheduling problems. Similarly, Ref. 32 applied the Tabu Search algorithm to the Quadratic Assignment Problem (QAP), showing its ability to efficiently explore the solution space and produce high-quality solutions compared to classical heuristics. Finally, Ref. 33 developed kernel-based nonlinear Support Vector Machine (SVM) models to address classification tasks involving non-linearly separable data, reporting significant improvements in classification accuracy across diverse real-world applications. In Ref. 34, develops new mathematical theorems to address the multi-criteria scheduling problem in three-machine flow shops, aiming to minimize both make span and the range of lateness. The findings show that applying these theorems provides efficient solutions and enhances job sequencing through dominance criteria while reducing computational complexity.

 The aim of this paper is to analyze the basic properties of work scheduling within a due-window with specified time periods, with a focus on finding an efficient solution to the problem. The paper structure is summarized as follows: presents some basic theories and concepts required in the research. The mathematical model for the problem $1\left|[d_{i}^{\left(1\right)},d_{i}^{\left(2\right)}]\right|F\left({\mathrm{ET}}_{\max},V_{\max}\right)$ is formulated. Also, presents some special cases. In addition, the dominance rules and relationships between functions are reviewed. Finally, summarizes the conclusions.

## Important notations

n : Number of jobs.

$p_{i}$ : Processing time of job i .

$d_{i}^{\left(1\right)}$ : The start time of Due-window of job i .

$d_{i}^{\left(2\right)}$ : The end time of Due-window of job i .

$s_{i}$ : Slack time of job i s.t. $s_{i}=d_{i}^{\left(1\right)}-p_{i}$ .

$C_{i}:$ Completion time of job i .

$E_{i}:$ Earliness value of the job i .

$T_{i}:$ Tardiness value of the job i .

$V_{i}$ : Late work value of the job i .

## Basic concepts of combinatorial optimization problems

In this paper, we shall use the following sequencing rules and concepts:

Theorem 1. ^35^ The problem of minimizing maximum earliness subject to no machine idle time, denoted as $1\|E_{\max},$ is solved by sequencing the jobs according to the Minimum Slack Time (MST)− rule, that is, in order of non-decreasing $s_{i}=d_{i}^{\left(1\right)}-p_{i}.$

Theorem 2. ^35^ The problem of minimizing maximum tardiness subject to no machine idle time, denoted as $1\|T_{\max}$ , is solved by sequencing the jobs according to the Earliest Due Date (EDD)− rule, that is, in order of non-decreasing $d_{i}^{\left(2\right)}.$

Definition 1. ^36^ In the Latest Processing Time (LPT)− rule: Sequencing all jobs in non-increasing order of the processing times $p_{i}$ i.e. $\left(\;p_{1}\geq p_{2}\geq \cdots \geq p_{n}\right).$

Definition 2. ^37^ The term “optimize” in a multi-objective decision making problem refers to a solution around which there is no way of improving any objective without worsening at least one other objective.

Definition 3. ^35^ A feasible schedule σ is Pareto optimal with respect to the objective functions $f_{1},f_{2},\ldots ,f_{n}$ if there is no feasible schedule π with $f_{i}\left(\pi \right)\leq f_{i}\left(\sigma \right)$ for i=1,…,n , where at least one of the inequalities is strict.

Definition 4. ^38^ A solution s dominates $s^{\prime }$ if the z=f(s) dominates $z^{\prime }=f\left(s^{\prime }\right)$ , this is, $f_{i}\left(s\right)\leq f_{i}\left(s^{\prime }\right)\;$ for all i and $f_{i}\left(s\right)<f_{i}\left(s^{\prime }\right)$ for at least one i .

Theorem 3. ^35^ The $1|\;|f_{\max}$ problem is solved as follows: while there are unassigned jobs, assign the job that has minimum cost when scheduled in the last unassigned position to that position.

### Lawler Algorithm (LA) ^37^:

Step (1): let N={1,…,n},ω=(∅) and M be the set of all jobs with no successors.

Step (2): let $j^{*}$ such that $f_{j^{*}}\left(\sum p_{i}\right)=\mathrm{Min}\left\{f_{j}\left(\sum p_{i}\right)\right\},j\in M.$

Set $N=N-\left\{j^{*}\right\}$ and sequence the job $j^{*}$ in the last position of ω. Modify M to represent the new set of scheduled jobs.

Step (3): If N=∅ stop, otherwise go to step (2).

We claim that:

$$E_{\max}^{*}=\text{Minimum}\;E_{\max}\;\mathrm{by}\;\text{MST‐rule}.$$

$$T_{\max}^{*}=\text{Minimum}\;T_{\max}\;\mathrm{by}\;\text{EDD‐rule}.$$

$$V_{\max}^{*}=\text{Minimum}\;V_{\max}\;\mathrm{by}\;\mathrm{LA}.$$

## Model formulation

 Let N={1,2,…,n} be the set of n jobs that must be processed by a machine. Each job i has a processing time $p_{i}$ , and the due-window of job i is specified by a pair of non-negative real numbers $\left[d_{i}^{\left(1\right)},d_{i}^{\left(2\right)}\right]$ such that $d_{i}^{\left(1\right)}\leq d_{i}^{\left(2\right)}$ for i=1,2,…,n . Initially, all of the jobs are available to be processed by the machine and it starts processing without interrupted, and requires $p_{i}$ units of time to complete its processing. Thus, a schedule for the machine can be completely specified by giving the sequence in which the jobs are processed. For a given schedule *σ*, $C_{i}$ = $C_{i}$ ( *σ*) denotes the completion time of job i , $E_{i}=\max\left\{0,d_{i}^{\left(1\right)}-C_{i}\right\}$ is the earliness value of job $J_{i}$ , $T_{i}=\max\left\{0,C_{i}-d_{i}^{\left(2\right)}\right\}\;$ is the tardiness value of job $J_{i}$ and $V_{i}=\min\left\{T_{i},p_{i}\right\}$ is the late work of job $J_{i}$ and $D_{i}=d_{i}^{\left(2\right)}-d_{i}^{\left(1\right)}\;$ is the due-window size of job $J_{i}$ . For the slack due-window method, the due-window starting time $d_{i}^{\left(1\right)}\;$ and the due-window completion time $d_{i}^{\left(2\right)}\;$ for job $J_{i}$ are defined as:

$$d_{i}^{\left(1\right)}=p_{i}+q^{\left(1\right)}$$ (1)

$$d_{i}^{\left(2\right)}=p_{i}+q^{\left(2\right)}$$ (2)

respectively. Note that $p_{i}\;$ is the processing time of job $J_{i}$ . Since $q^{\left(1\right)}$ and $q^{\left(2\right)}$ are two job-independent constants, that satisfy $q^{\left(2\right)}>q^{\left(1\right)}$ , the window size $D_{i}=q^{\left(2\right)}-q^{\left(1\right)}$ is also a constant for all the jobs. ^39^

Now, let $E_{\max}=\max\left\{E_{i}\right\},\forall i=1,2,\ldots ,n$ be the maximum of earliness and $T_{\max}=\max\left\{T_{i}\right\},\forall i=1,2,\ldots ,n$ be the maximum of tardiness. Such that ${\mathrm{ET}}_{\max}=E_{\max}+T_{\max}$ be the maximum of earliness and tardiness $\left(f_{1}\right)$ , and let $V_{\max}=\max\left\{\min\left\{T_{i},p_{i}\right\}\right\}$ be the maximum of late work $\left(f_{2}\right).$

For a sequence of jobs $\sigma^{\prime }\in \sigma$ where σ is the set of all feasible solutions, the criteria $\left(f_{1}\right)$ and $\left(f_{2}\right)$ are computed follows as:

$$\begin{array}{cc} \begin{array}{c} \begin{array}{c} \mathrm{Min}\left\{\begin{array}{c} f_{1}\left(\sigma \right)={\mathrm{ET}}_{\max}\\ f_{2}\left(\sigma \right)=V_{\max}\; \end{array}\right\}\;\begin{array}{c} \left(3\right)\\ \left(4\right) \end{array}\;\\ s.t.\\ E_{i}=\left\{ \begin{array}{ccc} 0, & d_{i}^{\left(1\right)}\leq C_{i} & i=1,2,\ldots ,n\\ d_{i}^{\left(1\right)}-C_{i}, & d_{i}^{\left(1\right)}>C_{i} & i=1,2,\ldots ,n \end{array}\right., \end{array}\\ T_{i}=\left\{ \begin{array}{ccc} 0, & d_{i}^{\left(2\right)}\geq C_{i} & i=1,2,\ldots ,n\\ C_{i}-d_{i}^{\left(2\right)}, & d_{i}^{\left(2\right)}<C_{i} & i=1,2,\ldots ,n \end{array}\right., \end{array}\;\\ V_{i}=\left\{ \begin{array}{ccc} 0, & d_{i}^{\left(2\right)}\geq C_{i} & i=1,2,\ldots ,n\\ C_{i}-d_{i}^{\left(2\right)}, & d_{i}^{\left(2\right)}<C_{i}<d_{i}^{\left(2\right)}+p_{i} & i=1,2,\ldots ,n\\ p_{i}, & d_{i}^{\left(2\right)}+p_{i}\leq C_{i} & i=1,2,\ldots ,n \end{array}\right.\\ E_{\max}=\max_{i}\left\{E_{i}\right\}, & i=1,2,\ldots ,n\;\\ T_{\max}=\max_{i}\left\{T_{i}\right\}, & i=1,2,\ldots ,n\;\\ V_{\max}=\max_{i}\left\{V_{i}\right\}=\max_{i}\left\{\min\left\{T_{i},p_{i}\right\}\right\}, & i=1,2,\ldots ,n\; \end{array}\}\;\left(P\right)$$

The problem $1\left|[d_{i}^{\left(1\right)},d_{i}^{\left(2\right)}]\right|F\left({\mathrm{ET}}_{\max},V_{\max}\right)$ is NP -hard since the problem $1\|{\mathrm{ET}}_{\max},$ is NP-hard.

## Special cases

Case (1). If $d_{i}^{\left(1\right)}>C_{i},\forall i\in N$ then the MST− sequence is an ES for the problem (P) , as shown in  Table 1.

Proof. Since $d_{i}^{\left(1\right)}>C_{i},$ for all i∈N, we obtain that all the jobs are early i.e. $(\;T_{i}=0,\forall i\in N.$ So $T_{\max}=0)$ , and $(V_{i}=0,\forall i\in N$ . So $V_{\max}=0)$ , and $E_{i}=\left\{d_{i}^{\left(1\right)}-C_{i},\forall i\in N\right\}.$

So $E_{\max}=\max\left\{d_{i}^{\left(1\right)}-C_{i}\right\},\forall i\in N,$ and the problem (P) can be state as: $F\left({\mathrm{ET}}_{\max},V_{\max}\right)=F\left(E_{\max},0\right)=F\left(\max\left\{d_{i}^{\left(1\right)}-C_{i}\right\},0\right)$ .

Since the MST− sequence yields optimal solution for $\;E_{\max},$ it follows that the MST− sequence is an ES for the problem (P) .

Case (2). If $d_{i}^{\left(2\right)}<C_{i},\forall i\in N\backslash \left\{1\right\}\;\left(C_{1}\in \left[d_{1}^{\left(1\right)},d_{1}^{\left(2\right)}\right]\right)$ for the problem (P) , then we obtain three cases:

(a) If $d_{i}^{\left(2\right)}<C_{i}<d_{i}^{\left(2\right)}+p_{i}$ ∀i∈N\{1} , then the EDD -sequence is an ES .
(b) If $d_{i}^{\left(2\right)}+p_{i}\leq C_{i}$ ∀i∈N\{1} , then the EDD -sequence is an ES .
(c) If $d_{i}^{\left(2\right)}<C_{i}<d_{i}^{\left(2\right)}+p_{i}$ or $d_{i}^{\left(2\right)}+p_{i}\leq C_{i},i\in N\backslash \left\{1\right\},$ for any different jobs i,j∈N\{1} , s.t. $p_{i}<p_{j}$ and $d_{i}^{\left(2\right)}<d_{j}^{\left(2\right)}$ , then the EDD‐ sequence is an ES , as shown in Table 1.

Proof (a). Since $d_{i}^{\left(2\right)}<C_{i}$ except $\left(C_{1}\in \left[d_{1}^{\left(1\right)},d_{1}^{\left(2\right)}\right]\right),\forall i\in N\backslash \left\{1\right\}$ this means that all the jobs are not early i.e. $(E_{i}=0,\forall i\in N$ . So $E_{\max}=0)$ ,

For $f_{1}$ problem: ${\mathrm{ET}}_{\max}=T_{\max}=\max\left\{T_{i}\right\}=\max\left\{\max\left\{C_{i}-d_{i}^{\left(2\right)},0\right\}\right\}=\max\left\{C_{i}-d_{i}^{\left(2\right)}\right\},i\in N.$

Therefore, $F\left({\mathrm{ET}}_{\max},V_{\max}\right)=F\left(T_{\max}^{*},V_{\max}\right)=\left(\max\left\{C_{i}-d_{i}^{\left(2\right)}\right\},V_{\max}\right),i\in N$ .

For $f_{2}$ problem: If $d_{i}^{\left(2\right)}<C_{i}<d_{i}^{\left(2\right)}+p_{i}$ except $\left(C_{1}\in \left[d_{1}^{\left(1\right)},d_{1}^{\left(2\right)}\right]\right),\forall i\in N\backslash \left\{1\right\}$ , then $V_{i}=C_{i}-d_{i}^{\left(2\right)},\left(V_{\max}=\max\left\{V_{i}\right\}=\max\left\{C_{i}-d_{i}^{\left(2\right)}\right\}=T_{\max}\right),i\in N\backslash \left\{1\right\}.$

So, the problem (P) can be state as the following:

$$F\left({\mathrm{ET}}_{\max},V_{\max}\right)=F\left(T_{\max},V_{\max}\right)=F\left(T_{\max},T_{\max}\right)=\left(\max\left\{C_{i}-d_{i}^{\left(2\right)}\right\},\max\left\{C_{i}-d_{i}^{\left(2\right)}\right\}\right),\forall i\in N\;$$

Now, since the EDD‐ sequence gives optimal solution for $T_{\max},$ then the EDD‐ sequence is an ES .

Proof (b). Suppose that σ be the EDD‐ sequence.

Now, since $\;d_{i}^{\left(2\right)}+p_{i}\leq C_{i}⟹d_{i}^{\left(2\right)}<C_{i},\left(C_{1}\in \left[d_{1}^{\left(1\right)},d_{1}^{\left(2\right)}\right]\right),\forall i\in N\backslash \left\{1\right\}$ ,

which implies all the jobs are not early i.e. $(E_{i}=0,\forall i\in N$ . So $E_{\max}=0)$ ,

For $f_{1}$ problem: ${\mathrm{ET}}_{\max}=T_{\max}=\max\left\{T_{i}\right\}=\max\left\{\max\left\{C_{i}-d_{i}^{\left(2\right)},0\right\}\right\}=\max\left\{C_{i}-d_{i}^{\left(2\right)}\right\},i\in N.$

For $f_{2}$ problem: If $d_{i}^{\left(2\right)}+p_{i}\leq C_{i}\left(C_{1}\in \left[d_{1}^{\left(1\right)},d_{1}^{\left(2\right)}\right]\right),\forall i\in N\backslash \left\{1\right\},$ then $V_{i}=p_{i},$ and $p_{i}\leq T_{i},\forall i\in N\backslash \left\{1\right\},$ i.e. $\left(V_{\max}=\max\left\{\min\left\{T_{i},p_{i}\right\}\right\}=\max\left\{p_{i}\right\}=p_{\max},\forall i\in N\backslash \left\{1\right\}\right)$ .

So, the problem (P) can be presented as the following:

$$F\left({\mathrm{ET}}_{\max},V_{\max}\right)=F\left(T_{\max},V_{\max}\right)=F\left(T_{\max},\max\left\{p_{i}\right\}\right)=\left(\max\left\{C_{i}-d_{i}^{\left(2\right)}\right\},p_{\max}\right),i\in N.$$

Therefore, the sequence σ is an ES by Theorem (2).

Proof (c). Suppose that σ be the EDD‐ sequence.

Since $d_{i}^{\left(2\right)}<C_{i},\left(C_{1}\in \left[d_{1}^{\left(1\right)},d_{1}^{\left(2\right)}\right]\right),\forall i\in N\backslash \left\{1\right\}$ , this leads to all the jobs are not early i.e. $(\;E_{i}=0,\forall i\in N$ . So $E_{\max}=0)$ ,

For $f_{1}$ problem: ${\mathrm{ET}}_{\max}=T_{\max}=\max\left\{T_{i}\right\}=\max\left\{\max\left\{C_{i}-d_{i}^{\left(2\right)},0\right\}\right\}=\max\left\{C_{i}-d_{i}^{\left(2\right)}\right\},i\in N$ .

For $f_{2}$ problem: If $d_{i}^{\left(2\right)}<C_{i}<d_{i}^{\left(2\right)}+p_{i}$ for $\left(C_{1}\in \left[d_{1}^{\left(1\right)},d_{1}^{\left(2\right)}\right]\right),\forall i\in N\backslash \left\{1\right\}$ , then $V_{i}=C_{i}-d_{i}^{\left(2\right)},\left(C_{1}\in \left[d_{1}^{\left(1\right)},d_{1}^{\left(2\right)}\right]\right),\forall i\in N\backslash \left\{1\right\}$ and if $d_{i}^{\left(2\right)}+p_{i}\leq C_{i},\forall i\in N\backslash \left\{1\right\}$ , then $V_{i}=p_{i}$ , i.e. $\left(V_{\max}=\max\left\{V_{i}\right\}=\max\left\{\min\left\{\{C_{i}-d_{i}^{\left(2\right)}\},p_{i}\right\}\right\}\right),$ i∈N\{1} .

Therefore, the problem (P) can be introduced as the following:

$$F\left({\mathrm{ET}}_{\max},V_{\max}\right)=F\left(T_{\max},V_{\max}\right)=\left(\max_{i\in N}\left\{C_{i}-d_{i}^{\left(2\right)}\right\},\max_{i\in N\backslash \left\{1\right\}}\left\{\min_{i\in N\backslash \left\{1\right\}}\left\{\{C_{i}-d_{i}^{\left(2\right)}\},p_{i}\right\}\right\}\right).$$

Thus, the sequence σ is an ES by Theorem (2).

Case (3). If we have a $\sigma^{\prime }$ be any sequence of jobs such that $d_{i}^{\left(1\right)}\leq C_{i}\leq d_{i}^{\left(2\right)},\forall i\in N$ , then σ is an ES for the problem (P) , as shown in example (1).

Proof. Suppose that $\sigma^{\prime }$ be any sequence of jobs such that $d_{i}^{\left(1\right)}\leq C_{i}\leq d_{i}^{\left(2\right)},\forall i\in N,$ this means all the jobs JIT of due-windows.

For $f_{1}$ problem: $(E_{i}=0,\forall i\in N$ , so $E_{\max}=0)$ and $(T_{i}=0,\forall i\in N$ , so $T_{\max}=0)$ which yields $\;{\mathrm{ET}}_{\max}=0$ .

For $f_{2}\;$ problem: $(V_{i}=0,\forall i\in N$ , so $V_{\max}=0)$ .

Thus, the problem (P) can be written as the following:

$F\left({\mathrm{ET}}_{\max},V_{\max}\right)=\left(0,0\right)$ . So, any sequence σ with the condition $d_{i}^{\left(1\right)}\leq C_{i}\leq d_{i}^{\left(2\right)},\forall i\in N,$ is an ES for the problem (P) .

Proposition 1. If MST‐ rule, EDD‐ rule, LA and LPT‐ sequence are identical in one sequence, then this sequence gives only one ES for problem (P) , as shown in  Table 1.

Proof. Suppose that σ=MST=EDD=LA=LPT have the same sequence,

Since the MST‐ rule ensures the minimum value of $E_{\max}$ , schedule σ is an optimal for $E_{\max}$ according to Theorem (1). In addition, by applying the EDD‐ rule, $T_{\max}$ is minimum, which confirms that σ is optimal for $T_{\max}$ based on Theorem (2). Furthermore, the LA guarantees the minimization of $V_{\max}$ , which making σ optimal for $V_{\max}$ as established in Theorem (3).

To establish the uniqueness of schedule σ , let μ represent any arbitrary schedule. Then it holds that:

$E_{\max}\left(\sigma =\mathrm{MST}\right)\leq E_{\max}\left(\mu \right)$ , $T_{\max}\left(\sigma =\mathrm{EDD}\right)\leq T_{\max}\left(\mu \right)$ and $V_{\max}\left(\sigma =\mathrm{LA}\right)\leq V_{\max}\left(\mu \right)$ . Consequently, for the problem (P) the combines solution $\left({\mathrm{ET}}_{\max}\left(\sigma \right),V_{\max}\left(\sigma \right)\right)$ strictly dominates the solution $\left({\mathrm{ET}}_{\max}\left(\mu \right),V_{\max}\left(\mu \right)\right)$ .

Case (4). In the common due-windows for the problem (P) , if $d^{\left(1\right)}\geq C_{n}$ and we have the LPT‐ sequence then $F\left({\mathrm{ET}}_{\max},V_{\max}\right)=\left(d^{\left(1\right)}-C_{1},0\right)\;$ and the MST‐ sequence is ES , as shown in  Table 1.

Proof. Suppose that σ be LPT‐ sequence and MST‐ sequence.

Now, since $d^{\left(1\right)}>C_{i},\forall i\in N,$ this means that all the jobs are early. $(T_{i}=0$ and $V_{i}=0,\forall i\in N$ . So $T_{\max}=0$ and $V_{\max}=0)$ , respectively.

Since we have common due-windows, this means $\left[d_{i}^{\left(1\right)},d_{i}^{\left(2\right)}\right]=\left[d^{\left(1\right)},d^{\left(2\right)}\right],\forall i\in N.$

$E_{i}=d^{\left(1\right)}-C_{i},\forall i\in N,E_{\max}=\max\left\{\max\left\{d^{\left(1\right)}-C_{i},0\right\}\right\}=\max\left\{d^{\left(1\right)}-C_{i}\right\}=d^{\left(1\right)}-C_{1}.$ So, the MST‐ sequence gives optimal solution for $E_{\max}$ by Theorem (1).

Thus, the problem (P) can be written as the following:

$$F\left({\mathrm{ET}}_{\max},V_{\max}\right)=F\left(E_{\max},0\right)=\left(d^{\left(1\right)}-C_{1},0\right).$$

Hence, the sequence σ is ES for the problem ( *P*).

Lemma (1). The ES for $F\left({\mathrm{ET}}_{\max},V_{\max}\right)=\left(d^{\left(1\right)}-C_{1},0\right)$ , where $d^{\left(1\right)}\geq C_{n}$ be $\left(d^{\left(1\right)}-p_{\max},0\right)$ .

Proof. It is clear by Definition (1).

Case (5). For the problem (P) with the common due-window $\left[d_{i}^{\left(1\right)},d_{i}^{\left(2\right)}\right]=\left[d^{\left(1\right)},d^{\left(2\right)}\right],\forall i\in N$ , if $d^{\left(2\right)}<C_{i},\left(C_{1}\in \left[d^{\left(1\right)},d^{\left(2\right)}\right]\right),\forall i\in N\backslash \left\{1\right\}$ , then conclude three cases:

(a) If $d^{\left(2\right)}<C_{i}<d^{\left(2\right)}+p_{i},\left(C_{1}\in \left[d^{\left(1\right)},d^{\left(2\right)}\right]\right),\forall i\in N\backslash \left\{1\right\}$ , then $F\left({\mathrm{ET}}_{\max},V_{\max}\right)=\left(C_{n}-d^{\left(2\right)},C_{n}-d^{\left(2\right)}\right)$ .
(b) If $d^{\left(2\right)}+p_{i}\leq C_{i},\left(C_{1}\in \left[d^{\left(1\right)},d^{\left(2\right)}\right]\right),\forall i\in N\backslash \left\{1\right\}$ , then $F\left({\mathrm{ET}}_{\max},V_{\max}\right)=\left(C_{n}-d^{\left(2\right)},p_{\max}\right)$ .
(c) If $d^{\left(2\right)}<C_{i}<d^{\left(2\right)}+p_{i}$ or $d^{\left(2\right)}+p_{i}\leq C_{i}$ , ∀i∈N\{1}, then $F\left({\mathrm{ET}}_{\max},V_{\max}\right)=\left(C_{n}-d^{\left(2\right)},\max_{i\in N\backslash \left\{1\right\}}\left\{\min_{i\in N\backslash \left\{1\right\}}\left\{\{C_{i}-d^{\left(2\right)}\},p_{i}\right\}\right\}\right)$ , as shown in Table 1.

Proof (a). Let $d^{\left(2\right)}<C_{i},\left(C_{1}\in \left[d^{\left(1\right)},d^{\left(2\right)}\right]\right),\forall i\in N\backslash \left\{1\right\},$ it follows that all the jobs are not early.

For $f_{1}$ problem: $E_{i}=0,\forall i\in N$ , therefore $E_{\max}=0$ ,

$$T_{i}=C_{i}-d^{\left(2\right)},\forall i\in N,\text{therefore}\;T_{\max}=\max\left\{T_{i}\right\}=\max\left\{\max\left\{C_{i}-d^{\left(2\right)},0\right\}\right\}=\max\left\{C_{i}-d^{\left(2\right)}\right\}=C_{n}-d^{\left(2\right)}.$$

For $f_{2}$ problem: $V_{i}=\min\left\{T_{i},p_{i}\right\}=T_{i},\forall i\in N\backslash \left\{1\right\}$ , i.e. $V_{\max}=\max\left\{V_{i}\right\}=\max\left\{T_{i}\right\}=T_{\max}=C_{n}-d^{\left(2\right)}$ .

Therefore, the problem (P) can introduced by:

$$F\left({\mathrm{ET}}_{\max},V_{\max}\right)=F\left(T_{\max},V_{\max}\right)=\left(C_{n}-d^{\left(2\right)},T_{\max}\right)=\left(C_{n}-d^{\left(2\right)},C_{n}-d^{\left(2\right)}\right).$$

Proof (b). If $d^{\left(2\right)}+p_{i}\leq C_{i},\left(C_{1}\in \left[d^{\left(1\right)},d^{\left(2\right)}\right]\right),\forall i\in N\backslash \left\{1\right\}$ , then all the jobs are not early.

For $f_{1}$ problem: $E_{\max}=0$ and $T_{\max}=C_{n}-d^{\left(2\right)}$ .

For $f_{2}$ problem: $V_{i}=\min\left\{T_{i},p_{i}\right\}=p_{i},\forall i\in N\backslash \left\{1\right\}$ , i.e. $V_{\max}=\max\left\{V_{i}\right\}=\max\left\{p_{i}\right\}=p_{\max}$ .

Therefore, the problem (P) can be formulated as:

$$F\left({\mathrm{ET}}_{\max},V_{\max}\right)=F\left(T_{\max},V_{\max}\right)=\left(C_{n}-d^{\left(2\right)},p_{\max}\right).$$

Proof (c). If $d^{\left(2\right)}<C_{i}<d^{\left(2\right)}+p_{i}$ or $d^{\left(2\right)}+p_{i}\leq C_{i},i\in N\backslash \left\{1\right\}$ .

For $f_{1}$ problem: $E_{\max}=0$ and $T_{\max}=C_{n}-d^{\left(2\right)}$ .

For $f_{2}$ problem: $V_{\max}=\max_{i\in N\backslash \left\{1\right\}}\left\{V_{i}\right\}=\max_{i\in N\backslash \left\{1\right\}}\left\{\min_{i\in N\backslash \left\{1\right\}}\left\{\{C_{i}-d_{i}^{\left(2\right)}\},p_{i}\right\}\right\}$ , ∀i∈N .

Therefore, the problem (P) can be written as:

$$F\left({\mathrm{ET}}_{\max},V_{\max}\right)=F\left(T_{\max},V_{\max}\right)=F\left(T_{\max},V_{\max}\right)=\left(C_{n}-d^{\left(2\right)},\max_{i\in N\backslash \left\{1\right\}}\left\{\min_{i\in N\backslash \left\{1\right\}}\left\{\{C_{i}-d^{\left(2\right)}\},p_{i}\right\}\right\}\right).$$

Case (6). For the problem (P) , if we have common processing time $p_{i}=p,\forall i\in N\;$ with the condition $d_{n}^{\left(1\right)}\geq C_{n}=\mathrm{np},\forall i\in N$ and we have the MST‐ sequence then this sequence is an ES and $F\left({\mathrm{ET}}_{\max},V_{\max}\right)=\left(d_{n}^{\left(1\right)}-\mathrm{np},0\right)$

Proof. Suppose that σ be MST‐ sequence which yields $\left(s_{1}\leq s_{2}\leq \cdots \leq s_{n}\right)=\left(d_{1}^{\left(1\right)}-p\leq d_{2}^{\left(1\right)}-p\leq \cdots \leq d_{n}^{\left(1\right)}-p\right)$ .

Assume $d_{i}^{\left(1\right)}\geq C_{n}=\mathrm{np},$ for all i∈N, this implies that all the jobs are early such that $T_{i}=0$ and $V_{i}=0,\forall i\in N$ , which gives $T_{\max}=0$ and $V_{\max}=0$ .

For each job i∈N the earliness is $E_{i}=d_{i}^{\left(1\right)}-C_{i}=d_{i}^{\left(1\right)}-\mathrm{ip}$ . Thus, $E_{\max}=\max\left\{d_{i}^{\left(1\right)}-C_{i},0\right\}=\max\left\{d_{i}^{\left(1\right)}-\mathrm{ip}\right\}=d_{n}^{\left(1\right)}-\mathrm{np}$ .

Therefore, the problem (P) is presented by:

$$F\left({\mathrm{ET}}_{\max},V_{\max}\right)=\left(E_{\max},0\right)=\left(\max_{i}\left\{d_{i}^{\left(1\right)}-\mathrm{ip}\right\},0\right)=\left(d_{n}^{\left(1\right)}-\mathrm{np},0\right).$$

By Theorem (1) this shows that the sequence σ is an ES for the problem (P) .

Case (7). For the problem (P) , if we have common processing time $p_{i}=p$ and $d_{i}^{\left(2\right)}<C_{i}=\mathrm{ip},\forall i\in N\backslash \left\{1\right\},\left(p\in \left[d_{1}^{\left(1\right)},d_{1}^{\left(2\right)}\right]\right)$ , then we have three cases:

(a) If $d_{i}^{\left(2\right)}<C_{i}<d_{i}^{\left(2\right)}+p,,\forall i\in N\backslash \left\{1\right\}$ and we have the EDD‐ sequence then this sequence is an ES and $F\left({\mathrm{ET}}_{\max},V_{\max}\right)=\left(\mathrm{np}-d_{\max}^{\left(2\right)},\mathrm{np}-d_{\max}^{\left(2\right)}\right)$ .
(b) If $d_{i}^{\left(2\right)}+p\leq C_{i},\forall i\in N\backslash \left\{1\right\}\;$ and we have the EDD‐ sequence, then this sequence is an ES and $F\left({\mathrm{ET}}_{\max},V_{\max}\right)=\left(\mathrm{np}-d_{\max}^{\left(2\right)},p\right)$ .
(c) If $d_{i}^{\left(2\right)}<C_{i}<d_{i}^{\left(2\right)}+p$ or $d_{i}^{\left(2\right)}+p\leq C_{i},i\neq 1$ and we have the EDD‐ sequence then this sequence is an ES and $\;F\left({\mathrm{ET}}_{\max},V_{\max}\right)=\left(\mathrm{np}-d_{\max}^{\left(2\right)},\max_{i\in N\backslash \left\{1\right\}}\left\{\min_{i\in N\backslash \left\{1\right\}}\left\{\{C_{i}-d_{i}^{\left(2\right)}\},p\right\}\right\}\right)$ .

Proof (a). Since $p_{i}=p,\forall i\in N\;\left(i.e.C_{n}=\sum_{i=1}^{n}p_{i}=\sum_{i=1}^{n}p=\mathrm{np}\right)$ , suppose that σ be an EDD‐ sequence which yields $\left(d_{1}^{\left(2\right)}\leq d_{2}^{\left(2\right)}\leq \ldots \leq d_{n}^{\left(2\right)}\right)$ , and since $d_{i}^{\left(2\right)}<C_{i}=\mathrm{ip},\forall i\in N\backslash \left\{1\right\}\;$ , that gives all the jobs are not early.

For $f_{1}$ problem: $E_{i}=0,\forall i\in N,\text{so}\;E_{\max}=0$ and $T_{i}=C_{i}-d_{i}^{\left(2\right)}=\mathrm{ip}-d_{i}^{\left(2\right)},\forall i\in N,\text{so}\;T_{\max}=\max\left\{\max\left\{\mathrm{ip}-d_{i}^{\left(2\right)},0\right\}\right\}=\max\left\{\mathrm{ip}-d_{i}^{\left(2\right)}\right\}=\mathrm{np}-d_{\max}^{\left(2\right)}$ ,

For $f_{2}$ problem: Since $d_{i}^{\left(2\right)}<C_{i}<d_{i}^{\left(2\right)}+p,\forall i\in N\backslash \left\{1\right\},$ this means $V_{i}=\min\left\{T_{i},p_{i}\right\}=T_{i},\forall i\in N$ , therefore $V_{\max}=\max\left\{V_{i}\right\}=\max\left\{T_{i}\right\}=\max\left\{\mathrm{ip}-d_{i}^{\left(2\right)}\right\}=\mathrm{np}-d_{\max}^{\left(2\right)}$ .

Therefore, the problem (P) can be state as:

$$F\left({\mathrm{ET}}_{\max},V_{\max}\right)=F\left(T_{\max},V_{\max}\right)=\left(\max\left\{\mathrm{ip}-d_{i}^{\left(2\right)}\right\},\max\left\{\mathrm{ip}-d_{i}^{\left(2\right)}\right\}\right)=\left(\mathrm{np}-d_{\max}^{\left(2\right)},\mathrm{np}-d_{\max}^{\left(2\right)}\right).$$

Hence, the sequence σ is ES for the problem ( *P*).

Proof (b). Since $p_{i}=p,\forall i\in N,\left(i.e.C_{n}=\sum_{i=1}^{n}p_{i}=\sum_{i=1}^{n}p=\mathrm{np}\right)$ ,

Suppose that σ is an EDD‐ sequence which yields $\left(d_{1}^{\left(2\right)}\leq d_{2}^{\left(2\right)}\leq \ldots \leq d_{n}^{\left(2\right)}\right)\;$ and since $d_{i}^{\left(2\right)}<C_{i}=\mathrm{ip},\forall i\in N\backslash \left\{1\right\}$ , this implies all the jobs are not early.

For $f_{1}\;$ problem: $E_{i}=0,\forall i\in N,\text{so}\;E_{\max}=0$ and $T_{i}=C_{i}-d_{i}^{\left(2\right)}=\mathrm{ip}-d_{i}^{\left(2\right)},\forall i\in N,\text{so}\;T_{\max}=\max\left\{\max\left\{\mathrm{ip}-d_{i}^{\left(2\right)},0\right\}\right\}=\max\left\{\mathrm{ip}-d_{i}^{\left(2\right)}\right\}=\mathrm{np}-d_{\max}^{\left(2\right)},$

For $f_{2}\;$ problem: since $d_{i}^{\left(2\right)}+p\leq C_{i},\forall i\in N\backslash \left\{1\right\},$ which means $V_{i}=\min\left\{T_{i},p\right\}=p,\forall i\in N,\text{so}\;V_{\max}=\max\left\{V_{i}\right\}=\max\left\{p\right\}=p.$

So, we can be state the problem (P) is stated as:

$$F\left({\mathrm{ET}}_{\max},V_{\max}\right)=F\left(T_{\max},V_{\max}\right)=\left(\max\left\{\mathrm{ip}-d_{i}^{\left(2\right)}\right\},p\right)=\left(\mathrm{np}-d_{\max}^{\left(2\right)},p\right).$$

Hence, the sequence σ is an ES for the problem (P) .

Proof (c). Since $p_{i}=p,\forall i\in N,\left(i.e.C_{n}=\sum_{i=1}^{n}p_{i}=\sum_{i=1}^{n}p=\mathrm{np}\right)$ , suppose that σ is an EDD‐ sequence which yields $\left(d_{1}^{\left(2\right)}\leq d_{2}^{\left(2\right)}\leq \cdots \leq d_{n}^{\left(2\right)}\right),$ and since $d_{i}^{\left(2\right)}<C_{i}=\mathrm{ip},\forall i\in N\backslash \left\{1\right\}$ , this means all the jobs are not early.

For $f_{1}$ problem: $E_{i}=0,\forall i\in N,$ therefore $E_{\max}=0$ and $T_{i}=C_{i}-d_{i}^{\left(2\right)}=\mathrm{ip}-d_{i}^{\left(2\right)},\forall i\in N,$ so $T_{\max}=\max\left\{\max\left\{\mathrm{ip}-d_{i}^{\left(2\right)},0\right\}\right\}=\max\left\{\mathrm{ip}-d_{i}^{\left(2\right)}\right\}=\mathrm{np}-d_{\max}^{\left(2\right)}$ .

For $f_{2}$ problem: if $d_{i}^{\left(2\right)}<C_{i}<d_{i}^{\left(2\right)}+p$ or $d_{i}^{\left(2\right)}+p\leq C_{i},i\neq 1,$ then $V_{i}=\min\left\{T_{i},p\right\},\forall i\in N\backslash \left\{1\right\},$ so $V_{\max}=\max_{i\in N\backslash \left\{1\right\}}\left\{\min_{i\in N\backslash \left\{1\right\}}\left\{\{C_{i}-d_{i}^{\left(2\right)}\},p\right\}\right\}$ .

Thus, the problem (P) can be written as:

$$F\left({\mathrm{ET}}_{\max},V_{\max}\right)=F\left(T_{\max},V_{\max}\right)=\left(\mathrm{np}-d_{\max}^{\left(2\right)},\max_{i\in N\backslash \left\{1\right\}}\left\{\min_{i\in N\backslash \left\{1\right\}}\left\{\{C_{i}-d_{i}^{\left(2\right)}\},p\right\}\right\}\right).$$

Hence, the sequence σ is an ES for the problem (P) .

Case (8). For the problem ( *P*), If $p_{i}=p,\forall i\in N$ and $\left[d_{i}^{\left(1\right)},d_{i}^{\left(2\right)}\right]=\left[d^{\left(1\right)},d^{\left(2\right)}\right]$ with the condition $d^{\left(1\right)}>C_{i}\;$ , ∀i∈N, then $F\left({\mathrm{ET}}_{\max},V_{\max}\right)=\left(d^{\left(1\right)}-C_{1},0\right)$ and any sequence is an ES .

Proof: Suppose that σ be any sequence, if $d^{\left(1\right)}>C_{i},$ for every job i∈N then this implies all the jobs are early. Consequently, $T_{i}=0\;\text{and}\;V_{i}=0,\forall i\in N.$ Thus, both the maximum tardiness and the maximum late of work are equal to zero. For each job i , the earliness is given by: $E_{i}=d^{\left(1\right)}-C_{i}=d^{\left(1\right)}-\mathrm{ip},\forall i\in N$ , so ${\mathrm{ET}}_{\max}=E_{\max}=\max\left\{d^{\left(1\right)}-C_{i},0\right\}=d^{\left(1\right)}-\min_{i\in N}C_{i}$ . Since all processing times are identical $\left(p_{i}=p\right)$ , the earliest completion time corresponds to the first scheduled job. Hence $\min_{i\in N}C_{i}=C_{1}$ . It follows that $E_{\max}=d^{\left(1\right)}-C_{1}$ , $V_{\max}=0.$

Therefore, we can be state the problem (P) as a following:

$$F\left({\mathrm{ET}}_{\max},V_{\max}\right)=\left(E_{\max},0\right)=\left(d^{\left(1\right)}-C_{1},0\right).$$

Hence, the sequence σ is an ES for the problem (P).

Lemma (2). The ES for $F\left({\mathrm{ET}}_{\max},V_{\max}\right)=\left(d^{\left(1\right)}-C_{1},0\right)$ , where $d^{\left(1\right)}\geq C_{n}$ be $\left(d^{\left(1\right)}-p,0\right)$ .

Proof: It is clear from Lemma (1).

Case (9). For the problem (P) , if $p_{i}=p$ and $\left[d_{i}^{\left(1\right)},d_{i}^{\left(2\right)}\right]=\left[d^{\left(1\right)},d^{\left(2\right)}\right]$ , ∀i∈N and $d^{\left(1\right)}\geq p$ (i.e. if the jobs have identical of the processing times and due windows) with $C_{1}=p\in \left[d^{\left(1\right)},d^{\left(2\right)}\right]$ then we have three subcases:

(a) If $d^{\left(2\right)}<C_{i}<d^{\left(2\right)}+p,\forall i\in N\backslash \left\{1\right\}$ then $\left({\mathrm{ET}}_{\max},V_{\max}\right)=\left(\mathrm{np}-d^{\left(2\right)},\mathrm{np}-d^{\left(2\right)}\right)$ and any sequence is an ES .
(b) If $d^{\left(2\right)}+p\leq C_{i},,\forall i\in N\backslash \left\{1\right\}$ , then $\left({\mathrm{ET}}_{\max},V_{\max}\right)=\left(\mathrm{np}-d^{\left(2\right)},p\right)$ and any sequence is an ES .
(c) If $d^{\left(2\right)}<C_{i}<d^{\left(2\right)}+p$ or $d^{\left(2\right)}+p\leq C_{i},i\in N\backslash \left\{1\right\}$ , then $\left({\mathrm{ET}}_{\max},V_{\max}\right)=\left(\mathrm{np}-d^{\left(2\right)},\max_{i\in N\backslash \left\{1\right\}}\left\{\min_{i\in N\backslash \left\{1\right\}}\left\{\{C_{i}-d^{\left(2\right)}\},p\right\}\right\}\right)$ and any sequence is an ES .

Proof (a): Suppose that σ be any sequence, if $d^{\left(2\right)}<C_{i},$ for all i∈N , it follows that all the jobs are not early. Consequently, $E_{i}=0,$ for all i∈N, so $E_{\max}=0$

Since $p_{i}=p,$ for all i∈N, we have $\;C_{n}=\sum_{i=1}^{n}p_{i}=\sum_{i=1}^{n}p=\mathrm{np}$ ,

The tardiness of jobs $T_{i}=C_{i}-d^{\left(2\right)}=\mathrm{ip}-d^{\left(2\right)},$ for all i∈N , $\;\text{so}\;T_{\max}=\max\left\{\max\left\{\mathrm{ip}-d^{\left(2\right)},0\right\}\right\}=\max\left\{\mathrm{ip}-d^{\left(2\right)}\right\}=\mathrm{np}-d^{\left(2\right)}$ . So ${\mathrm{ET}}_{\max}=T_{\max}=\max\left\{\max\left\{\mathrm{ip}-d^{\left(2\right)},0\right\}\right\}=\max\left\{\mathrm{ip}-d^{\left(2\right)}\right\}=\mathrm{np}-d^{\left(2\right)}$ .

Now, since $d_{i}^{\left(2\right)}<C_{i}<d_{i}^{\left(2\right)}+p,$ for all job i∈N\{1}, we get $V_{i}=\min\left\{T_{i},p_{i}\right\}=T_{i},\forall i\in N$ , so $V_{\max}=\max\left\{V_{i}\right\}=\max\left\{T_{i}\right\}=\max\left\{\mathrm{ip}-d^{\left(2\right)}\right\}=\mathrm{np}-d^{\left(2\right)}$ .

Therefore, the problem (P) can be introduced as:

$$\left({\mathrm{ET}}_{\max},V_{\max}\right)=\left(T_{\max},V_{\max}\right)=\left(\max\left\{\mathrm{ip}-d_{i}^{\left(2\right)}\right\},\max\left\{\mathrm{ip}-d_{i}^{\left(2\right)}\right\}\right)=\left(\mathrm{np}-d^{\left(2\right)},\mathrm{np}-d^{\left(2\right)}\right).$$

Hence, the sequence σ is an ES for the problem (P) .

Proof (b). Suppose that σ be any sequence, if the condition $d^{\left(2\right)}<C_{i}=\mathrm{ip},\forall i\in N\backslash \left\{1\right\}$ , it follows that no jobs are early. Consequently, $E_{i}=0,$ for all $i\in N,\text{so}\;E_{\max}=0$ .

Since $p_{i}=p,\forall i\in N,\left(i.e.C_{n}=\sum_{i=1}^{n}p_{i}=\sum_{i=1}^{n}p=\mathrm{np}\right)$ ,

The tardiness of jobs $T_{i}=C_{i}-d^{\left(2\right)}=\mathrm{ip}-d^{\left(2\right)},$ for all i∈N, so $\;T_{\max}=\max\left\{\max\left\{\mathrm{ip}-d^{\left(2\right)},0\right\}\right\}=\max\left\{\mathrm{ip}-d^{\left(2\right)}\right\}=\mathrm{np}-d^{\left(2\right)},$

Since $d^{\left(2\right)}+p\leq C_{i},$ for all job i∈N\{1}, which implies $V_{i}=\min\left\{T_{i},p\right\}=p,\forall i\in N,$ so $\;V_{\max}=\max\left\{V_{i}\right\}=\max\left\{p\right\}=p$

Therefore, the problem (P) can be introduced by:

$$\left({\mathrm{ET}}_{\max},V_{\max}\right)=\left(T_{\max},V_{\max}\right)=\left(\max\left\{\mathrm{ip}-d_{i}^{\left(2\right)}\right\},\max\left\{p\right\}\right)=\left(\mathrm{np}-d^{\left(2\right)},p\right).$$

Hence, the sequence σ is an ES for the problem (P) .

Proof (c). Suppose that σ be any sequence, if $d^{\left(2\right)}<C_{i}=\mathrm{ip},\forall i\in N\backslash \left\{1\right\}$ , it follows that jobs are not early. Thus, $E_{i}=0,\forall i\in N,$ so $\;E_{\max}=0$

Since $p_{i}=p,$ for all i∈N, we have $C_{n}=\sum_{i=1}^{n}p_{i}=\sum_{i=1}^{n}p=\mathrm{np}$ ,

Now, the tardiness of jobs $T_{i}=C_{i}-d^{\left(2\right)}=\mathrm{ip}-d^{\left(2\right)},\forall i\in N$ , so $T_{\max}=\max\left\{\max\left\{\mathrm{ip}-d^{\left(2\right)},0\right\}\right\}=\max\left\{\mathrm{ip}-d^{\left(2\right)}\right\}=\mathrm{np}-d^{\left(2\right)}$ .

And if $d^{\left(2\right)}<C_{i}<d^{\left(2\right)}+p\;$ or $d^{\left(2\right)}+p\leq C_{i},i\in N\backslash \left\{1\right\},$ then $V_{i}=\min\left\{T_{i},p\right\}i\in N\backslash \left\{1\right\},$ so $V_{\max}=\max_{i\in N\backslash \left\{1\right\}}\left\{\min_{i\in N\backslash \left\{1\right\}}\left\{\{C_{i}-d^{\left(2\right)}\},p\right\}\right\}.$

Therefore, the problem (P) can be written as:

$$\left({\mathrm{ET}}_{\max},V_{\max}\right)=\left(\mathrm{np}-d^{\left(2\right)},\max_{i\in N\backslash \left\{1\right\}}\left\{\min_{i\in N\backslash \left\{1\right\}}\left\{\{C_{i}-d^{\left(2\right)}\},p\right\}\right\}\right).$$

Hence, for the problem (P) , the sequence σ is an ES .

Lemma (3). The ES for $F\left({\mathrm{ET}}_{\max},V_{\max}\right)=\left(C_{n}-d^{\left(2\right)},\max_{i\in N\backslash \left\{1\right\}}\left\{\min_{i\in N\backslash \left\{1\right\}}\left\{\{C_{i}-d^{\left(2\right)}\},p\right\}\right\}\right)$ , where $d^{\left(2\right)}<C_{i}<d^{\left(2\right)}+p$ or $C_{i}<d^{\left(2\right)}+p,i\in N$ be $\left({\mathrm{ET}}_{\max},V_{\max}\right)=\left(\mathrm{np}-d^{\left(2\right)},\min\left\{\mathrm{np}-d^{\left(2\right)},p\right\}\right)$ .

Proof: It is clear by $C_{n}=\sum_{i=1}^{n}p_{i}=\sum_{i=1}^{n}p=\mathrm{np}$ .

In Appendix 1,  Table 1 presents an example for each special case, specifying the conditions that must be satisfied in each case, and illustrating the validity of the results by comparing them with those obtained using the Complete Enumeration Method (CEM).

**Table 1.** Compare between the special cases and CEM.

Cases	Conditions	$\left[p_{i};d_{i}^{\left(1\right)};d_{i}^{\left(2\right)}\right]$	Sequence	*F*( *ET*_*max*_, *V*_*max*_)	*CEM*
**1**	$d_{i}^{\left(1\right)}>C_{i},\forall i\in N$	$\left[\begin{array}{cccc} 2 & 4 & 6 & 8\\ 2 & 8 & 15 & 22\\ 5 & 10 & 20 & 25 \end{array}\right]$	MST=(1,2,3,4)	(3,0)	(3,0)
2−a	$d_{i}^{\left(2\right)}<C_{i},\forall i\in N\backslash \left\{1\right\}$ & $d_{i}^{\left(2\right)}<C_{i}<d_{i}^{\left(2\right)}+p_{i}$	$\left[\begin{array}{cccc} 2 & 4 & 6 & 8\\ 2 & 4 & 8 & 9\\ 3 & 5 & 10 & 15 \end{array}\right]$	EDD=(1,2,3,4)	(5,5)	(5,5) (15,4) (17,3)
2−b	$d_{i}^{\left(2\right)}<C_{i},\forall i\in N\backslash \left\{1\right\}$ & $d_{i}^{\left(2\right)}+p_{i}\leq C_{i}$	$\left[\begin{array}{cccc} 4 & 2 & 3 & 6\\ 4 & 2 & 4 & 6\\ 6 & 3 & 5 & 8 \end{array}\right]$	EDD=(2,3,1,4)	(7,6)	(7,6) (12,5) (9,4) (10,3)
2−c	$d_{i}^{\left(2\right)}<C_{i}$ ,∀i∈N\{1} , $d_{i}^{\left(2\right)}<C_{i}<d_{i}^{\left(2\right)}+p_{i}$ & $d_{j}^{\left(2\right)}+p_{j}\leq C_{j}$	$\left[\begin{array}{cccc} 4 & 6 & 8 & 10\\ 4 & 6 & 9 & 12\\ 5 & 8 & 10 & 15 \end{array}\right]$	EDD=(1,2,3,4)	(13,10)	(13,10) (18,8) (21,6) (20,7)
**3**	$d_{i}^{\left(1\right)}\leq C_{i}\leq d_{i}^{\left(2\right)},\forall i\in N$	$\left[\begin{array}{cccc} 4 & 6 & 8 & 10\\ 4 & 8 & 10 & 15\\ 5 & 10 & 20 & 30 \end{array}\right]$	σ=(1,2,3,4)	(0,0)	(0,0)
**Proposition 1**	MST=EDD=LA=LPT	$\left[\begin{array}{cccc} 8 & 6 & 4 & 2\\ 8 & 6 & 5 & 3\\ 10 & 12 & 15 & 20 \end{array}\right]$	σ=(1,2,3,4)	(3,3)	(3,3)
**4**	$\left[d^{\left(1\right)},d^{\left(2\right)}\right],\forall i\in N$ & $d^{\left(1\right)}\geq C_{n}$	$\left[\begin{array}{cccc} 8 & 6 & 4 & 2\\ 20 & 20 & 20 & 20\\ 25 & 25 & 25 & 25 \end{array}\right]$	MST=(1,2,3,4)	(12,0)	(12,0)
5−a	$\left[d^{\left(1\right)},d^{\left(2\right)}\right]$ , $d^{\left(2\right)}<C_{i},\forall i\in N\backslash \left\{1\right\}$ & $d^{\left(2\right)}<C_{i}<d^{\left(2\right)}+p_{i}\;\left(C_{n}-d^{\left(2\right)},C_{n}-d^{\left(2\right)}\right)$	$\left[\begin{array}{cccc} 2 & 3 & 2 & 3\\ 3 & 3 & 3 & 3\\ 8 & 8 & 8 & 8 \end{array}\right]$	σ=(1,2,3,4)	(2,2)	(2,2)
5−b	$\left[d^{\left(1\right)},d^{\left(2\right)}\right]$ , $d^{\left(2\right)}<C_{i},\forall i\in N\backslash \left\{1\right\}$ & $d^{\left(2\right)}+p_{i}\leq C_{i}\;\left(C_{n}-d^{\left(2\right)},p_{\max}\right)$	$\left[\begin{array}{cccc} 6 & 4 & 2 & 2\\ 6 & 6 & 6 & 6\\ 8 & 8 & 8 & 8 \end{array}\right]$	σ=(1,2,3,4)	(6,2)	(6,2)
5−c	$\left[d^{\left(1\right)},d^{\left(2\right)}\right]$ , $d^{\left(2\right)}<C_{i},\forall i\in N\backslash \left\{1\right\}$ , $d^{\left(2\right)}<C_{i}<d^{\left(2\right)}+p_{i}$ & $d^{\left(2\right)}+p_{j}\leq C_{j}\;\left(C_{n}-d^{\left(2\right)},\max_{i\in N\backslash \left\{1\right\}}\left\{\min_{i\in N\left\{1\right\}}\left\{\{C_{i}-d^{\left(2\right)}\},p_{i}\right\}\right\}\right)$	$\left[\begin{array}{cccc} 6 & 4 & 3 & 3\\ 6 & 6 & 6 & 6\\ 10 & 10 & 10 & 10 \end{array}\right]$	σ=(1,2,3,4)	(6,3)	(6,3)
**6**	$p_{i}=p$ & $d_{i}^{\left(1\right)}\geq C_{n}=\mathrm{np},\forall i\in N\;\left(d_{n}^{\left(1\right)}-\mathrm{np},0\right)$	$\left[\begin{array}{cccc} 2 & 2 & 2 & 2\\ 2 & 6 & 10 & 12\\ 3 & 8 & 12 & 15 \end{array}\right]$	MST=(1,2,3,4)	(4,0)	(4,0)
7−a	$p_{i}=p$ , $d_{i}^{\left(2\right)}<C_{i}=\mathrm{ip}$ & $d_{i}^{\left(2\right)}<C_{i}<d_{i}^{\left(2\right)}+p,\forall i\in N\backslash \left\{1\right\}\;\left(\mathrm{np}-d_{\max}^{\left(2\right)},\mathrm{np}-d_{\max}^{\left(2\right)}\right)$	$\left[\begin{array}{cccc} 4 & 4 & 4 & 4\\ 4 & 5 & 5 & 8\\ 5 & 6 & 10 & 13 \end{array}\right]$	EDD=(1,2,3,4)	(3,3)	(3,3)
7−b	$p_{i}=p$ , $d_{i}^{\left(2\right)}<C_{i}=\mathrm{ip}$ & $d_{i}^{\left(2\right)}+p\leq C_{i},\forall i\in N\backslash \left\{1\right\}\;\left(\mathrm{np}-d_{\max}^{\left(2\right)},p\right)$	$\left[\begin{array}{cccc} 2 & 2 & 2 & 2\\ 2 & 2 & 2 & 3\\ 5 & 4 & 3 & 5 \end{array}\right]$	EDD=(3,2,1,4)	(3,2)	(3,2)
7−c	$p_{i}=p$ , $d_{i}^{\left(2\right)}<C_{i}=\mathrm{ip}$ , $d_{i}^{\left(2\right)}<C_{i}<d_{i}^{\left(2\right)}+p,\forall i\in N\backslash \left\{1\right\}$ & $d_{j}^{\left(2\right)}+p\leq C_{j}\;\left(\mathrm{np}-d_{\max}^{\left(2\right)},\max\left\{\min\left\{\mathrm{np}-d_{\max}^{\left(2\right)},p\right\}\right\}\right)$	$\left[\begin{array}{cccc} 4 & 4 & 4 & 4\\ 4 & 6 & 8 & 8\\ 5 & 6 & 7 & 10 \end{array}\right]$	EDD=(1,2,3,4)	(6,4)	(6,4)
**8**	$p_{i}=p$ , $\left[d^{\left(1\right)},d^{\left(2\right)}\right]$ & $d^{\left(1\right)}>C_{i},\forall i\in N\backslash \left\{1\right\}\;\left(d^{\left(1\right)}-C_{1},0\right)$	$\left[\begin{array}{cccc} 2 & 2 & 2 & 2\\ 10 & 10 & 10 & 10\\ 12 & 12 & 12 & 12 \end{array}\right]$	σ=(1,2,3,4)	(8,0)	(8,0)
9−a	$p_{i}=p$ , $\left[d^{\left(1\right)},d^{\left(2\right)}\right]\;d^{\left(1\right)}\geq p$ & $d^{\left(2\right)}<\mathrm{ip}<d^{\left(2\right)}+p,\forall i\in N\backslash \left\{1\right\}\;\left(\mathrm{np}-d^{\left(2\right)},\mathrm{np}-d^{\left(2\right)}\right)$	$\left[\begin{array}{cccc} 2 & 2 & 2 & 2\\ 2 & 2 & 2 & 2\\ 6 & 6 & 6 & 6 \end{array}\right]$	σ=(1,2,3,4)	(2,2)	(2,2)
9−b	$p_{i}=p$ , $\left[d^{\left(1\right)},d^{\left(2\right)}\right]$ , $d^{\left(1\right)}\geq p$ & $d^{\left(2\right)}+p\leq \mathrm{ip},\forall i\in N\backslash \left\{1\right\}\;\left(\mathrm{np}-d^{\left(2\right)},p\right)$	$\left[\begin{array}{cccc} 4 & 4 & 4 & 4\\ 4 & 4 & 4 & 4\\ 8 & 8 & 8 & 8 \end{array}\right]$	σ=(1,2,3,4)	(12,4)	(12,4)
9−c	$p_{i}=p$ , $\left[d^{\left(1\right)},d^{\left(2\right)}\right]$ , $d^{\left(1\right)}\geq p$ & $d^{\left(2\right)}<\mathrm{ip}<d^{\left(2\right)}+p,\forall i\in N\backslash \left\{1\right\}\;$ or $d^{\left(2\right)}+p\leq \mathrm{ip}\;\left(\mathrm{np}-d^{\left(2\right)},\max\left\{\min\left\{\mathrm{np}-d^{\left(2\right)},p\right\}\right\}\right)\;$	$\left[\begin{array}{cccc} 4 & 4 & 4 & 4\\ 4 & 4 & 4 & 4\\ 6 & 6 & 6 & 6 \end{array}\right]$	σ=(1,2,3,4)	(10,4)	(10,4)

## Dominance rules

DR1. For the problem (P) , if two adjacent jobs i,j∈σ satisfy the conditions $p_{i}\leq p_{j},d_{i}^{\left(1\right)}\leq d_{j}^{\left(1\right)}\;\text{and}\;d_{i}^{\left(2\right)}\leq d_{j}^{\left(2\right)}$ , then there exists an ES in which a job i is processed before job j .

Proof. Since $p_{i}\leq p_{j}\;$ and $\;d_{i}^{\left(1\right)}\leq d_{j}^{\left(1\right)}$ it follows that, $s_{i}=d_{i}^{\left(1\right)}-p_{i}\leq d_{j}^{\left(1\right)}-p_{j}=s_{j}$ . According to the MST‐ rule, this inequality leads to $E_{i}\leq E_{j}.$

Moreover, from the EDD‐ rule with the condition $d_{i}^{\left(2\right)}\leq d_{j}^{\left(2\right)}$ , we also obtain $T_{i}\leq T_{j}$ . Consequently, ${\mathrm{ET}}_{i}\leq {\mathrm{ET}}_{j},$ which means that there exists at least one ES in which job i precedes job j .

Now, if $V_{i}\leq V_{j}$ for some sequence where the job i precede j .

overwise if $V_{i}>V_{j}=\min\left\{T_{i},p_{i}\right\}>\min\left\{T_{j},p_{j}\right\}\;$ which means we have three subcases:

Case (1): If we have two adjacent jobs i,j∈σ satisfy the conditions $d_{i}^{\left(2\right)}<C_{i}<d_{i}^{\left(2\right)}+p_{i}$ and $d_{j}^{\left(2\right)}<C_{j}<d_{j}^{\left(2\right)}+p_{j}$ then $V_{i}>V_{j}=T_{i}>T_{j}$ which it contradiction with EDD‐ rule. So $V_{i}\leq V_{j}.$

Case (2): If we have two adjacent jobs i,j∈σ satisfy the conditions $d_{i}^{\left(2\right)}+p_{i}\leq C_{i}$ and $d_{j}^{\left(2\right)}+p_{j}\leq C_{j},$ then $V_{i}>V_{j}=p_{i}>p_{j}$ which it contradiction with the condition $\;p_{i}\leq p_{j}$ . So $V_{i}\leq V_{j}.$

DR 2. For the problem (P) , for two jobs i,j∈σ such that $p_{i}\leq p_{j}$ and $d_{i}^{\left(1\right)}\leq d_{j}^{\left(1\right)}$ , the job i is (JIT)i.e. completed within window $\;d_{i}^{\left(1\right)}\leq C_{i}\leq d_{i}^{\left(2\right)}$ and the job j is satisfy the condition $d_{j}^{\left(1\right)}>C_{j}$ then job 𝑖 precede job *j*.

Proof. From Case (3), if $p_{i}\leq p_{j}\;\text{and}\;d_{i}^{\left(1\right)}\leq d_{j}^{\left(1\right)}$ such that the job i is $\left(\mathrm{JIT}\right)\;\text{i.e.}\;d_{i}^{\left(1\right)}\leq C_{i}\leq d_{i}^{\left(2\right)},i\in \sigma$ , then from the criteria function (P) we get: since $E_{i}=0,T_{i}=0\;\text{and}\;V_{i}=0,i\in \sigma$ .

Now, for the job j with $d_{j}^{\left(1\right)}>C_{j},\left(j\neq 1\right)$ that means $E_{j}=d_{j}^{\left(1\right)}-C_{j}>0,j\in \sigma \;$ but $T_{j}=0,V_{j}=0$ .

So, $E_{i}<E_{j},\forall i,j\in \sigma$ and $p_{i}\leq p_{j}\;\text{and}\;d_{i}^{\left(1\right)}\leq d_{j}^{\left(1\right)}$ . Thus, the job i precede job j .

DR 3. For the problem (P) , consider two jobs i and j belonging to a schedule σ . Assume that the following conditions hold $\;p_{i}\leq p_{j}$ and $d_{i}^{\left(2\right)}\leq d_{j}^{\left(2\right)}$ . Job i is completed within its Just-In-Time (JIT) window, that is, $d_{i}^{\left(1\right)}\leq C_{i}\leq d_{i}^{\left(2\right)}$ Conversely, job j is finished after the end of its due window but still before the maximum permissible delay, i.e., $d_{j}^{\left(2\right)}<C_{j}<d_{j}^{\left(2\right)}+p_{j}.$ Under these assumptions, there exists an ES in which job 𝑖 precede job *j*.

Proof. Based on Case (3), job i is completed exactly on time because its completion occurs within its (JIT) window. Therefore, the earliness, tardiness, and late of work values are all zero: $E_{i}=0,T_{i}=0\;\text{and}\;V_{i}=0,i\in \sigma$ .

Since job j with the condition $d_{j}^{\left(2\right)}<C_{j}<d_{j}^{\left(2\right)}+p_{j},\left(j\neq 1\right)$ , finishes after its latest due window but before the maximum tolerable delay, it incurs a positive tardiness penalty: $T_{j}=C_{j}-d_{j}^{\left(2\right)}>0$ and $V_{j}=\min\left\{T_{j},p_{j}\right\}=T_{j},j\in \sigma \;$ but $E_{j}=0$ .

Job i has zero tardiness and late of work values, while job j has positive ones. Considering that, $T_{i}<T_{j},$ and $V_{i}<V_{j}\forall i,j\in \sigma$ and $p_{i}\leq p_{j}$ . Thus, the job i precede job j .

DR 4. Within the framework of the (P) problem, consider two jobs i and j belonging to a schedule σ , where (j≠1) . Assume that $p_{i}\leq p_{j}$ and $d_{i}^{\left(2\right)}\leq d_{j}^{\left(2\right)}.$ Job i is completed within its valid due window, i.e., $d_{i}^{\left(1\right)}\leq C_{i}\leq d_{i}^{\left(2\right)},$ whereas job j is completed after its maximum permissible completion time, that is $d_{j}^{\left(2\right)}+p_{j}\leq C_{j}$ . Under these conditions, there exists an ES in which job i precedes job j .then job *i* precede job *j*.

Proof. According to Case (3), when job i satisfies $p_{i}\leq p_{j}\text{and}\;d_{i}^{\left(2\right)}\leq d_{j}^{\left(2\right)}$ and its completion time lies within its due window, it is classified as a (JIT) job with $d_{i}^{\left(1\right)}\leq C_{i}\leq d_{i}^{\left(2\right)},i\in \sigma$ . Therefore, its earliness, tardiness, and late of work measures are all zero: $E_{i}=0,T_{i}=0\;\text{and}\;V_{i}=0,i\in \sigma$ .

In contrast, job j with $d_{j}^{\left(2\right)}<C_{j}\;$ and $d_{j}^{\left(2\right)}+p_{j}\leq C_{j}$ (j≠1) completes beyond the upper bound of its allowable window, representing a significant delay. Consequently, its tardiness and penalty functions can be expressed as: $T_{j}=C_{j}-d_{j}^{\left(2\right)}>0$ and $V_{j}=\min\left\{T_{j},p_{j}\right\}=p_{j},\;j\in \sigma$ but $E_{j}=0$ .

Since job i incurs no tardiness or penalty, while job j does $\left(T_{i}<T_{j},\text{and}\;V_{i}<V_{j}\forall i,j\in \sigma \;\right)$ , and given that $p_{i}\leq p_{j}\;\text{and}\;d_{i}^{\left(2\right)}\leq d_{j}^{\left(2\right)}$ , it follows that job i should be scheduled before job j in the efficient sequence (ES) . Thus, the job i precede job j .

DR 5. Within the framework of the (P) problem, consider two jobs i and j belonging to a schedule σ , where j≠1 , and both have equal processing times, i.e., $p_{i}=p_{j}=p$ . Suppose that $d_{i}^{\left(1\right)}\leq d_{j}^{\left(1\right)}$ such that the job i is in due window (JIT) with the condition $d_{i}^{\left(1\right)}\leq \mathrm{ip}\leq d_{j}^{\left(2\right)}$ while job j is completed earlier than its earliest due time, i.e., satisfies $d_{j}^{\left(1\right)}>C_{j}=\mathrm{jp}.$ Under these conditions, there exists an (ES) in which job i precedes job j.

Proof. According to Case (3), if job i meets the condition $d_{i}^{\left(1\right)}\leq \mathrm{ip}\leq d_{i}^{\left(2\right)}$ and is completed within its due window, it is classified as a Just-In-Time (JIT) job. Consequently, the earliness, tardiness, and late of work values of job i are all zero: $E_{i}=0,T_{i}=0\;\text{and}\;V_{i}=0,i\in \sigma$ .

Conversely, job j is completed earlier than its earliest allowable due window with $\;d_{j}^{\left(1\right)}>C_{j}$ , which results in an earliness penalty. Thus, its performance measures are: $E_{j}=d_{j}^{\left(1\right)}-C_{j}=d_{j}^{\left(1\right)}-\mathrm{jp}>0,T_{j}=0$ and $V_{j}=0,j\in \sigma \;\left(j\neq 1\right).$

Given that $d_{i}^{\left(1\right)}\leq d_{j}^{\left(1\right)}$ , it follows that the earliness value of job i is less than that of job j , i.e., $E_{i}\leq E_{j}$ . Therefore, from the objective function of the (p) problem, it is preferable to schedule job i before job j in the efficient sequence. Hence, under the given assumptions, an efficient schedule exists in which job i precedes job j , thereby reducing total earliness and improving the overall scheduling efficiency in the (P) environment.

DR 6. Within the framework of the (P) problem, consider two jobs i and j belonging to a schedule σ , where j≠1 , and both have equal processing times $p_{i}=p_{j}=p.$ Assume that $d_{i}^{\left(2\right)}\leq d_{j}^{\left(2\right)}$ . Job i is completed within its valid due window, i.e., $d_{i}^{\left(1\right)}\leq \mathrm{ip}\leq d_{i}^{\left(2\right)}$ , whereas job j is completed after the end of its due window but still within the permissible tolerance range, i.e., $d_{j}^{\left(2\right)}<C_{j}<d_{j}^{\left(2\right)}+p.$ Under these conditions, there exists an (ES) in which job i precedes job j.

Proof. According to Case (3), if job i satisfies $d_{i}^{\left(1\right)}\leq \mathrm{ip}\leq d_{i}^{\left(2\right)}$ and is completed within its due window, it is classified as a (JIT) job. Hence, its earliness, tardiness, and late of work values are all zero: $E_{i}=0$ , $T_{i}=0\;$ and $V_{i}=0,i\in \sigma$ .

For job j , since it finishes after its latest due time but still within the tolerance limit, it incurs a positive tardiness penalty. The tardiness and late of work values can be expressed as: $T_{j}=C_{j}-d_{j}^{\left(2\right)}=\mathrm{jp}-d_{j}^{\left(2\right)}>0$ and $V_{j}=T_{j}=C_{j}-d_{j}^{\left(2\right)}=\mathrm{jp}-d_{j}^{\left(2\right)}>0,\;j\in \sigma$ .

Given that $d_{i}^{\left(2\right)}\leq d_{j}^{\left(2\right)},$ it follows that the completion of job i results in lower tardiness and late of work values, i.e., $T_{i}<T_{j}\;$ and $V_{i}<V_{j}$ . Consequently, job i should be scheduled before job j in the efficient sequence to minimize the overall function in the (P) framework. Therefore, under the stated assumptions, an efficient schedule exists in which job i is executed prior to job j, ensuring minimal tardiness and improved schedule efficiency in the (P) problem.

DR 7. Within the framework of the (P) problem, consider two jobs i and j with equal processing times, $p_{i}=p_{j}=p$ , and assume that $\;d_{i}^{\left(2\right)}\leq d_{j}^{\left(2\right)}.$ Let job i be a (JIT) job, and suppose that job j is completed after its due window but still within its tolerance windows, i.e., $d_{i}^{\left(1\right)}\leq \mathrm{ip}\leq d_{i}^{\left(2\right)}$ and $d_{j}^{\left(2\right)}+p\leq C_{j}=\mathrm{jp},i,j\in \sigma ,\left(j\neq 1\right).$ Under these conditions, there exists an (ES) in which job i is scheduled before job j .

Proof. According to Case (3), if job i satisfies $d_{i}^{\left(1\right)}\leq \mathrm{ip}\leq d_{i}^{\left(2\right)}$ and is completed within its due window, it is classified as a (JIT) job. Consequently, its earliness, tardiness, and penalty values are all zero: $E_{i}=0$ , $T_{i}=0\;$ and $V_{i}=0,i\in \sigma$ . Job j , however, finishes after the end of its due window but still within the allowed tolerance. Its tardiness and late of work values are given by: $T_{j}=C_{j}-d_{j}^{\left(2\right)}=\mathrm{jp}-d_{j}^{\left(2\right)}>0\;$ and $V_{j}=p_{j}=p,\;j\in \sigma$ .

Since, $d_{i}^{\left(2\right)}\leq d_{j}^{\left(2\right)}$ , it follows that $T_{i}<T_{j}\;$ and $V_{i}<V_{j}$ . Therefore, job i should be scheduled before job j in the efficient sequence to minimize tardiness and late of work. Under the stated conditions, an efficient schedule exists in which job i precedes job j , ensuring better adherence to due windows and overall schedule efficiency in the (P) problem.

## Conclusion

The study examined the problem of scheduling jobs on a single machine within due-windows under multi-criteria objectives, where the focus was on minimizing the maximum earliness–tardiness together with the maximum late work. Through the development of mathematical formulations, special cases, and dominance rules, the work showed how classical sequencing rules such as MST,EDD,LPT and LA can be adapted to produce efficient solutions under specific conditions despite the NP-hardness of the problem.

However, the analysis is restricted to a deterministic single-machine setting with fixed due-window parameters. It does not capture more complex environments such as multiple machines, stochastic processing times, or dynamic job arrivals, which often arise in real-world production and service systems.

The results provide useful insights for just-in-time scheduling applications where penalties from earliness and tardiness have significant cost implications. In practice, the proposed rules and formulations can assist decision-makers in improving efficiency and reducing delays. Future research could extend this framework to more general scheduling contexts, integrate uncertainty factors, or develop heuristic and metaheuristic approaches to handle larger problem instances effectively.

Nevertheless, this study remains theoretical such that the practical implications of the proposed dominance rules in real-world scheduling systems were not analyzed. These aspects represent important directions for future work.

## Future work

In the future, the search can be extended to address additional cases by modifying the criteria or adding new constraints to the problem.

such as $1\left|[d_{i}^{\left(1\right)},d_{i}^{\left(2\right)}]\right|\left({\mathrm{ETV}}_{\max}\right)$ and $1\left|[d_{i}^{\left(1\right)},d_{i}^{\left(2\right)}],r_{i}\right|\left(\sum_{i=1}^{n}C_{i},{\mathrm{ET}}_{\max}\right)$ .

## Data Availability

The datasets supporting the findings of this article have been deposited in the Zenodo repository and are publicly available at the following link: https://doi.org/10.5281/zenodo.17587905 (Mohammed, 2025). The repository contains a collection of examples that compare the special cases studied in this work with the exhaustive enumeration method. Data are available under the terms of the Creative Commons Attribution 4.0 International license (CC-BY 4.0). This study adopts a secondary data analysis approach, systematically reviewing and integrating publicly available information obtained from peer-reviewed academic journals, credible investigative journalism sources, open-source intelligence platforms, technical documents, and international legal frameworks. No primary data collection, experimental procedures, or research involving human participants were conducted. All referenced materials are thoroughly cited in the References section of the manuscript and remain available through their original publishers. The authors declare that they have no relevant or material financial interests that relate to the research described in this paper. For data-related queries, contact doaha.a@s.uokerbala.edu.iq.
