# Supplementary material for: Single-Machine Scheduling with Multi-Criteria and Due- Windows
Source: F1000Res. 2026 Jan 12;15:47. [Version 1] doi: 10.12688/f1000research.173324.1 (PMC13463723; doi:10.12688/f1000research.173324.1)
Supplement: Peer review history

# Reviewer report: Reviewer response for version 1

**DOI:** 10.5256/f1000research.191129.r452641

**Published:** 2026-04-23

**Authors:** Faez Hassan Ali

**Affiliations:** 1 Mustansiriyah University, Bagdad, Iraq

- The abstract is too lengthy and can be significantly shortened.
- It is not recommended to include future work in the abstract.
- The researcher used the phrase "(Ref.1)." It would have been better to include the researcher's name and the year of the study, presenting the type of problem discussed, the solution method, and the results.
- Sources Ref. 28 (and Ref. 32, 33) are not related to the field of MSP. It would be preferable to include a historical study of sources working in the field of Multicriteri.
- In the paper outlines section, it is recommended to specify what the research covered in each section.
- In Theorem (3), does fmax refer to all max functions such as Tmax, Emax, etc.?
- It is recommended to explain why Lawler's method was used.
- Are q(1) and q(2) constant for all work? If so, then D1=D2=... = Dn, which is incorrect. Therefore, q(1) and q(2) should be written as qi(1) and qi(2). Please verify this.
- In the mathematical formulation and the rest of the research, sequence (i) is usually used for the machine and sequence (j) for the work.
- Tmax, Emax, Vmax ³ 0 should be added to the formulation of problem (P).
- It is preferable to place table (1) after the last case discussed, as it is illogical for the example to precede the problem.
- In case (2), paragraphs (a) and (b) can be combined.
- In case (2), what is the difference between paragraph (b) and the second part of paragraph (c)?
- The rule EDD can be referred to generally in case (2) instead of repeating it in paragraphs (a), (b), and (c).
- What is the purpose of LPT in prop (1) if EDDºMSTºLA? I think we don't need it.
- It is preferable to write EDDºMSTºLA instead of EDD=MST=LA.
- I believe that in case (6), it will be EDDºMST as long as p is constant.
- In lemma(3), the condition pi=p, di(1)=d(1), and di(2)=d(2) must be specified.
- It is preferable to assign a section number to the Dominance Rule section, as it is an important part.
- When writing a MSP, we have to write lines vertically (|), not diagonally (/).
- The research does not require a dataset because it is purely theoretical.
- It would have been preferable to support the research with the practical aspect (solution methods).

Is the work clearly and accurately presented and does it cite the current literature?

Yes

If applicable, is the statistical analysis and its interpretation appropriate?

Yes

Are all the source data underlying the results available to ensure full reproducibility?

Yes

Is the study design appropriate and is the work technically sound?

Partly

Are the conclusions drawn adequately supported by the results?

Partly

Are sufficient details of methods and analysis provided to allow replication by others?

Yes

Reviewer Expertise:

Operations Research, Combinatorial Optimization Problems.

I confirm that I have read this submission and believe that I have an appropriate level of expertise to confirm that it is of an acceptable scientific standard.

# Reviewer report: Reviewer response for version 1

**DOI:** 10.5256/f1000research.191129.r452643

**Published:** 2026-01-24

**Authors:** Iraq T. Abbas

**Affiliations:** 1 Mathematics, University of Baghdad, Baghdad, Iraq

The manuscript entitled *“Single-Machine Scheduling with Multi-Criteria and Due-Windows”* addresses a classical yet challenging problem in scheduling theory by investigating a single-machine environment with multiple performance criteria under due-window constraints. The authors formulate a comprehensive multi-criteria optimization model incorporating earliness, tardiness, and late work measures. By deriving a set of dominance rules and exploiting well-established sequencing principles, the study significantly reduces computational complexity while preserving optimality.

 The paper focuses on analytical rigor and theoretical soundness, supported by illustrative numerical examples and comparisons with complete enumeration. The proposed framework is relevant to practical applications such as Just-In-Time production systems and contributes meaningfully to the literature on exact scheduling methods.

Is the work clearly and accurately presented and does it cite the current literature?

Yes

If applicable, is the statistical analysis and its interpretation appropriate?

Not applicable

Are all the source data underlying the results available to ensure full reproducibility?

Yes

Is the study design appropriate and is the work technically sound?

Yes

Are the conclusions drawn adequately supported by the results?

Yes

Are sufficient details of methods and analysis provided to allow replication by others?

Yes

Reviewer Expertise:

Mathematics Operations Research

I confirm that I have read this submission and believe that I have an appropriate level of expertise to confirm that it is of an acceptable scientific standard.
